# Enabling Design of Secure IoT Systems with Trade-Off-Aware Architectural Tactics

**DOI:** 10.3390/s24227314

**Published:** 2024-11-15

**Authors:** Cristian Orellana, Francisco Cereceda-Balic, Mauricio Solar, Hernán Astudillo

**Affiliations:** 1 Departamento de Informática, Universidad Técnica Federico Santa María, Av. España 1680, Valparaíso 2390123, Chile; msolar@inf.utfsm.cl; 2 Centre for Environmental Technologies (CETAM) & Departament of Chemistry, Universidad Técnica Federico Santa María, Av. España 1680, Valparaíso 2390123, Chile; francisco.cereceda@usm.cl; 3 Instituto de Tecnología para la Innovación en Salud y Bienestar (ITiSB), Universidad Andrés Bello, Calle 1 Oriente 1180, Viña del Mar 2530959, Chile; hernan@acm.org

**Keywords:** internet of things, IoT cybersecurity, security tactics, architectural tactics, trade-offs, software architecture, STRIDE, ISO 25010

## Abstract

The increasing use of the Internet of Things (IoT) in homes and industry brings significant security and privacy challenges, while also considering trade-off for performance, energy consumption, and processing capabilities. Few explicit and specific guidelines exist to help architects in considering these trade-offs while designing secure IoT systems. This article proposes to address this situation by extending the well-known architectural tactics taxonomies with IoT-specific trade-offs; to preserving auditability, the trade-offs address the quality characteristics of the ISO 25010:2023 standard. The proposed technique and catalog are illustrated with the design of the Nunatak environmental monitoring system. The proposal was empirically validated with a controlled experiment, where a balanced mix of 12 novice and expert practitioners had to design a secure IoT Environmental Monitoring System; they used similar architectural tactics catalogs, with versus without trade-off information. Results suggest that having this information yield significant improvements in decision-making effectiveness (Precision) and usefulness (F1-Score), particularly benefiting less experienced designers. Wider adoption of trade-off-aware catalogs of architectural tactics will allow systematic, auditable design of secure IoT systems, and especially so by novice architects.

## 1. Introduction

The *Internet of Things (IoT)* is the interconnected network of physical devices, vehicles, home appliances, and other items embedded with sensors, software, and network connectivity that enables them to collect and exchange data. This concept involves the convergence of the digital and physical worlds, allowing for the seamless integration of information technology with the physical environment [1]. These “things” are equipped with sensing and actuation capabilities and, in some cases, programmable features. By leveraging these objects’ unique identification and sensing capabilities, information about their status can be controlled and manipulated from remote locations at any time. This level of connectivity allows for unprecedented levels of automation and control in several aspects of everyday life and industry [2,3].

IoT has seen rapid growth across several sectors [4,5], and it is estimated that by 2030, there will be 24.1 billion connected devices versus 500 million in 2003 [6]. This rapid expansion has brought significant challenges [7,8,9,10,11] for data integrity, user privacy, and operational continuity. Indeed, a 2020 survey in Japan, Canada, UK, Australia, USA, and France found that 63% of IoT consumers find these devices unsettling due to inadequate security measures [12], and other research has shown that 90% of consumers lack confidence in IoT cybersecurity [12]. Thus, personal data protection has given rise to regulations like *General Data Protection Regulation (GDPR)* [13], *Health Insurance Portability and Accountability Act of 1996 (HIPAA)* [14], and *California Consumer Privacy Act (CCPA)* [15], which require IoT systems to comply with strict data protection and privacy standards [16,17,18,19,20,21,22,23,24,25,26].

Designing secure IoT systems involves implementing security measures for hardware, software, communication, and updating mechanisms [27,28,29]. On the industry side, the lack of integrated development stacks supporting end-to-end IoT applications complicates collaboration among software architects and system designers working with different types of hardware and software [30], although there are some guidelines [7,31] and standards [32,33,34]. On the academia side, there is research on the design of secure IoT systems, but very little (mostly case studies) provides guidance for actual architects engaged in designing secure IoT systems [35]; specifically, there is limited evidence that traditional software architecture approaches are being systematically used to design secure IoT systems. This article extends architectural tactics for security, a design technique well-known in software architecture academic literature, with trade-off specifications for the impact of each tactic on quality attributes (as per the *ISO 25010* [36] standard). Trade-off information is crucial for architectural decision making, since a decision may affect several system properties besides the one intended to improve [37,38].

The proposal was validated with an experimental study involving twelve IT practitioners with varying seniority levels; it assessed the effectiveness, efficiency, usefulness, and accuracy of the trade-offs-aware Security Tactics catalog while designing a secure IoT system for an actual application for the Centre for Environmental Technologies (CETAM–UTFSM) for climate-science monitoring. Our findings show that providing IoT-specific trade-offs helped the subjects (junior and senior) to make better decisions (compared with a ground truth), and particularly helped junior architects improve recall of alternative solutions.

The key contributions are: (1) an approach to build scenario-specific trade-offs-aware architecture guidelines upon general-purpose, attribute-specific design knowledge; (2) an IoT-specific trade-offs-aware taxonomy of architectural tactics to support design of Secure IoT systems; and (3) empirical evidence that an IoT-specific trade-offs-aware taxonomy of architectural tactics offers effective, useful guidance for designing secure IoT systems.

The remainder of this article is structured as follows: Section 2 surveys previous work; Section 3 introduces the trade-off-aware catalog of security tactics; Section 4 illustrates its use with an actual case study; Section 5 presents the design and execution of an experimental study with practitioners; Section 6 discusses the experimental results; Section 7 addresses validity threats and their mitigation; and Section 8 summarizes and concludes.

## 2. Related Work

Software designers and architects designing secure IoT systems would benefit from guidance on design decisions regarding system structure and technologies. There are reference architectures for IoT (e.g., *ISO 30141* [34]), offering general guidance on architectural structure and underlying non-functional requirements; however, they do not offer guidance on the key design decisions to achieve specific quality attributes, nor how to consider tese decisions’ impact on other quality attributes. This is particularly relevant for IoT because many devices and systems have been designed for limited resources and deployed with very limited security capabilities, potentially creating serious security risks [7].

From a software architecture perspective, two main approaches have been proposed: patterns and tactics [39,40]. Furthermore, we will delve into reference architectures and threat modeling frameworks that provide alternative and complementary approaches for architecting robust security measures in IoT systems.

### 2.1. Security Patterns

Design patterns are reasoned solutions to recurrent problems [39]. Several authors have discussed security patterns and how they can be used to design secure systems. Thus, Fernandez [41] proposed in 2013 a catalog of security patterns, indicating how they can be systematically applied in different scenarios. A collaboration by security researchers and systems engineering researchers (Schumacher et al. [42]) introduced patterns to address security challenges in systems engineering; they provided illustrative examples of practical cases, but did not address IoT aspects.

Fernandez followed this up with a substantial body of work in numerous collaborations. Fernandez et al. [43] proposed an approach to evaluate system security using security patterns; since the method starts by identifying threats, they suggested that architectural tactics could enhance it, but did not assess their impact on system properties or their applicability to IoT systems.

Fernandez [44] introduced an IoT architecture pattern integrating cloud and fog computing to enhance data management and reduce latency. It safeguards data and communication through authentication, authorization, security logging, secure channels, and intrusion detection. However, it does not explore trade-offs with other quality attributes, limiting the understanding of interactions and impacts of competing requirements in an IoT ecosystem. Fernandez et al. [45] presented a pattern for secure implementations of Publish/Subscriber-based approaches in IoT systems. Although this pattern is widely applicable, especially since many IoT devices adopt such an architectural design, the discussion does not delve deeply into how its implementation affects other important quality attributes, such as performance. Fernandez and Yoshioka [46] argued for the importance of having a specific methodology, process, and conceptual security framework for designing secure systems; they presented a metamodel connecting the elements of a security problem and discussed architectural tactics as artifacts for designing secure systems, but did not integrate them into their metamodel, and did not provide IoT-specific guidelines. Fernandez et al. [47] presented a pattern for designing a secure *IoT Thing* from an architectural perspective, but did not discuss the trade-offs associated with their proposal. Fernandez et al. [48] explored the state-of-the-art patterns for designing secure IoT systems, but without specific details on building secure IoT systems and on the impact of decisions on quality attributes. Fernandez et al. [49] introduced the concept of *Abstract Security Patterns (ASPs)* as conceptual security mechanisms; they include functions to stop or mitigate a threat, comply with a regulation, or adhere to an institutional policy, but are not specific to IoT and neither describe their trade-offs.

Orellana et al. [50] proposed using architectural tactic categories from well-documented taxonomies to develop a pattern taxonomy focused on a specific quality attribute. While they thoroughly analyzed trade-offs related to the requirements of the illustrative case, they did not delve into the other quality attributes that IoT systems could have. In a later paper, they also introduced IoT patterns for designing a *Secure Sensor Node* [1] and a *Secure Actuator Node* [51], but without detailing how design decisions impact other attributes.

Washizaki et al. [52,53] performed a literature review looking for IoT design and architecture patterns, classified according to their level of abstraction and domain. They found a few articles that proposed IoT patterns and consider security, but they did not explore the matter of trade-offs.

Rajmohan et al. [35] performed a systematic literature review on IoT patterns and architectures. They concluded that IoT-specific patterns are relatively new, and that there is still a lack of documentation and adoption work.

Jamshidi et al. [54] evaluated the impact of security patterns on edge IoT applications, focusing on power consumption and CPU performance. Their results show that while these patterns improve security, they significantly increase resource usage. The authors called for more comprehensive security patterns that cover all aspects of IoT security and suggest improvements in scalability and response time. However, they do not propose specific design approaches, as their work mainly assesses existing patterns.

### 2.2. Security Tactics

*Architectural tactics* are alternative small-scale design decisions to satisfy specific quality attributes, and are organized in taxonomies [55]. Architectural tactics are the key design decisions that the system designer must make to ensure the system addressed the quality attributes of interest [50,56,57,58]. Architectural tactics are organized into taxonomies of solution alternatives [55,59]. Taxonomies of architectural tactics have been proposed for several quality attributes; e.g., *Availability*, *Performance* [55,60], and *Scalability* [61,62].

Bass et al. [63] proposed in 2003, and updated in later editions [55,64], a taxonomy of security tactics, with comprehensive security approach including applicability scenario, detailed tactics description, and a trade-offs overview. However, the limited description and lack of explicit relation to other specific quality attributes make it hard to apply this taxonomy to concrete IoT problems, which require decisions involving multiple quality attributes, besides considering IoT-specific limitations and restrictions.

Fernandez et al. [65] proposed a modified taxonomy, removing some tactics (as being policies or practices rather than design decisions) and proposing new ones, but did not consider trade-offs among attributes nor practical examples for IoT systems.

Rozanski and Woods [60] presented security tactics in their book, but did not organize them in a taxonomy or around a stimulus; thus, they are more akin to architectural principles (i.e., less specific conceptualizations) than to architectural tactics. Also, they did not elaborate on how to apply them in a specific IoT scenario.

Ryoo et al. [66] reorganized Bass’s security tactics [63] and established a new hierarchy of tactics. However, they did not provide a detailed taxonomy or consider trade-offs between other quality attributes.

Erder et al. [62] proposed security tactics not formally categorized, making it hard to evaluate alternative tactics, i.e., it is unclear whether each one helps to detect, resist, react to, or recover from an attack. Additionally, there is no information on interactions with other quality attributes, so the architect has few tools to evaluate the suitability of each tactic in a specific context (IoT or not).

Colesky et al. [67] proposed tactics for data protection in compliance with legal standards; they are limited to data protection, and not concerned with other security-related design decisions or security concerns, much less IoT implementations. Alshammari and Simpson [68] adopted this proposal and organized tactics in a hierarchical taxonomy, but without a detailed description or trade-off analysis; like [67], it does not address or illustrate how these tactics can be applied for IoT systems.

Pedraza-García et al. [69] presented an experimental approach to compare the effectiveness of tactics-based and pattern-based approaches in addressing security threats, and concluded that novices benefit more from a pattern-based approach since it provides a detailed guide to support their design. However, the illustrative example is not IoT-specific, although it does include some sensor-related software components.

Orellana et al. [70] introduced a systematic approach to address security threats with security tactics and their trade-offs as countermeasures; it showed the practical valuer with illustrative practical case, but did not not provide trade-off specifications for quality attributes beyond those outlined in the practical case, making it hard to extend it.

### 2.3. Reference Architectures

A *Reference Architecture* (RA) is a high-level representation that outlines a generic software architecture relevant to specific domains while omitting implementation details. A domain represents a specialized area with common characteristics across various applications, emphasizing functional aspects. IoT domains include healthcare, transportation, industry, smart cities, and autonomous vehicles. The literature frequently documents reference architectures for the IoT, and several secondary studies analyze and characterize these architectures in detail [53,71,72].

ISO/IEC 30141:2024 [34] is a widely extended reference architecture for IoT systems, emphasizing interoperability and the integration of devices and applications. It provides a framework for key components and their interactions but lacks specific implementation guidelines, leading to differing interpretations. Moreover, it does not adequately address trade-offs between quality requirements like security and performance, limiting the evaluation of design decisions in complex IoT systems.

Bashir et al. [73] presented a reference architecture for IoT smart buildings, showcasing how it enables the integration and management of IoT data, analytics, and control. However, it mainly identifies key components and their relationships without exploring alternative design solutions for specific issues. Its applicability is also limited to a specific domain, restricting its use in other areas.

Szmeja et al. [74] presented a reference architecture that addresses subsystems’ configurations, interrelations, and deployment strategies within an IoT ecosystem, outlining relevant protocols and interoperability mechanisms. However, it does not focus solely on security and lacks a critical analysis of trade-offs related to the system’s quality attributes.

### 2.4. Threat Modeling

Threat modeling is essential for IoT security due to the numerous vulnerabilities inherent in these complex systems. Techniques like *STRIDE* [75,76] and *DREAD* [77] help identify threats, such as phishing and data manipulation, but their effectiveness is hindered by the diversity of IoT devices and insufficient risk data. These techniques are often combined with other approaches to design secure systems.

Misuse patterns [78] are key in threat modeling, as they outline generic attacks and the vulnerabilities that enable them. These patterns offer countermeasures to prevent attacks and identify where forensic information can be found after an incident. Unfortunately, few IoT-specific misuse patterns exist [48]. Syed et al. [79] introduced a misuse pattern for an attack that exploits the security vulnerabilities of IoT devices, resulting in a *Denial of Service (DoS)*. The limited availability of IoT-specific misuse patterns underlines the need to develop more robust threat modeling tailored to the particularities of these interconnected systems. This will allow vulnerabilities to be addressed more effectively and improve security in the design of IoT solutions.

Table 1 summarizes related work proposing design patterns, architectural tactics, reference architectures, and architectural design elements through threat modeling. Some proposals from the related work have been excluded because they only utilize these architectural approaches to describe techniques or evaluations without providing an alternative solution for designing secure IoT systems. Similarly, secondary studies were not included in Table 1 as they divert focus from correctly identifying the research gap that is desired to be highlighted.

Table 1 highlights a significant gap in current research regarding designing secure IoT systems. Most proposed security patterns, reference architectures, and taxonomies of security tactics are not specifically tailored to the unique challenges and characteristics of the IoT environment. While some studies explore design patterns applicable to IoT, they generally lack the necessary adaptation to address the specific needs of this domain. Key factors such as the wide diversity of devices, communication over insecure networks, and limited computational resources are often overlooked. This lack of IoT-specific focus hinders the practical applicability of these proposals and limits their effectiveness in mitigating security risks unique to IoT environments.

Moreover, none of the reviewed approaches comprehensively addresses the trade-offs associated with implementing the proposed tactics or patterns, particularly in IoT. Designing secure IoT systems requires careful trade-offs to balance security with other quality attributes like efficiency, reliability, and scalability. Failing to analyze these trade-offs can lead to poor design choices that, although enhancing security, may undermine other crucial aspects of the system.

We propose developing an enriched taxonomy of security tactics tailored to IoT to address these gaps. This taxonomy will thoroughly analyze trade-offs concerning other relevant quality attributes based on the ISO 25010 standard. By incorporating these trade-offs, we aim to provide designers and architects with more comprehensive guidance for selecting security tactics that consider the diverse quality requirements of IoT systems.

## 3. A Trade-Offs-Aware Security Tactics Catalog

The trade-offs-aware security tactics catalog has two main components:

1. The *taxonomy of security tactics:* although several taxonomies of security tactics have been proposed, we adopt the latest version of the taxonomy by Bass et al. [55].
2. A *tabular description of IoT-specific trade-offs:* a newly created description of positive and negative impacts of each tactic on the typical quality attributes of an IoT system.

The following subsections describe the catalog parts in detail.

### 3.1. Security Tactics Taxonomy

**Figure 1:**
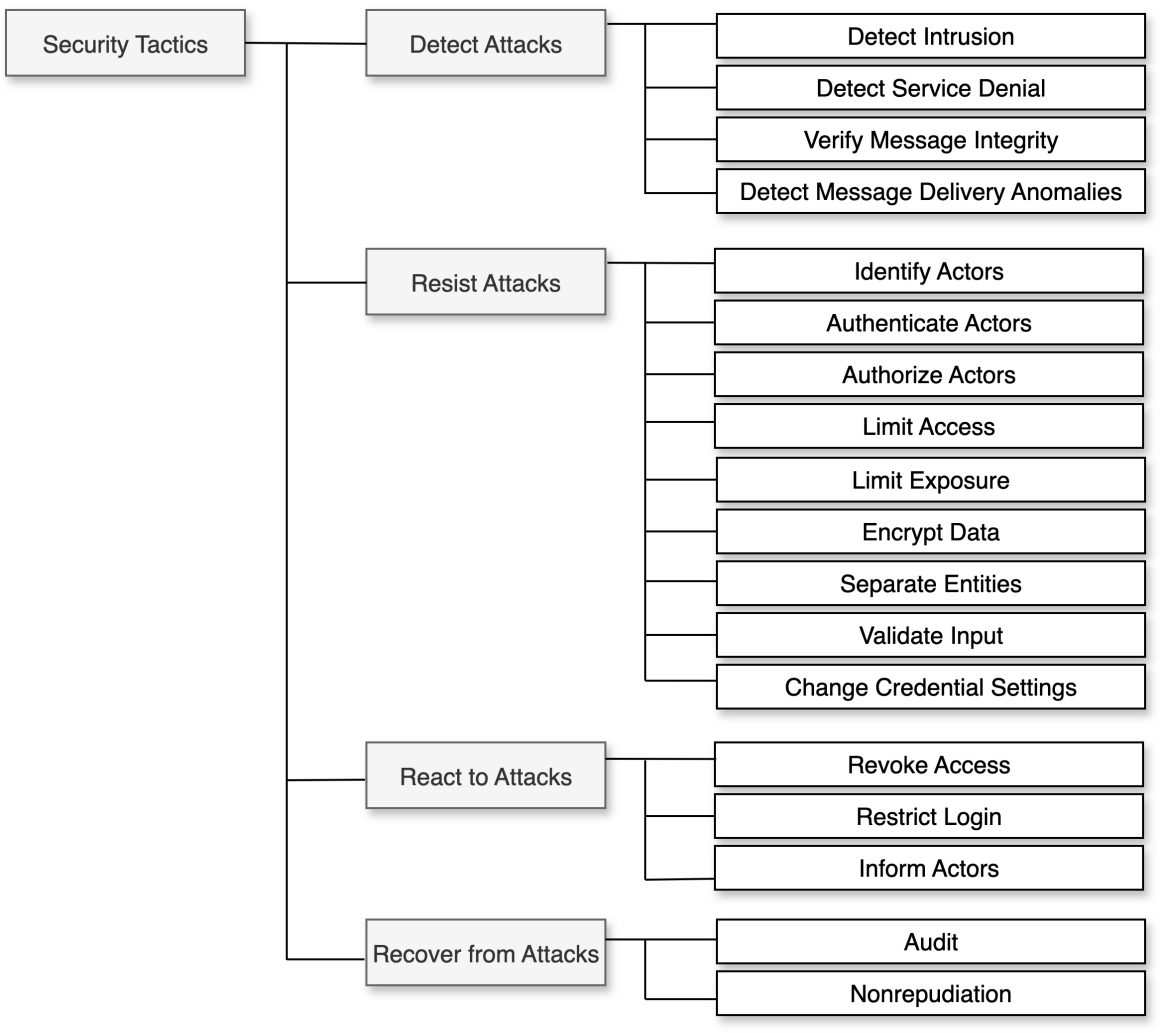
Security tactics taxonomy.

Figure 1 shows security tactics organized around the four key *design time* decisions that an architect must make to provide a software system with capabilities to address security concerns *at run time*: (1) how to *Detect Attacks*, i.e., early detection of situations that could compromise the security of a system; (2) how to *Resist Attacks*, i.e., mitigation measures to effectively manage a security issue; (3) how to *React to Attacks*, i.e., ability to respond to a potential attack; and (4) how to *Recover from Attacks*, i.e., ability to recover from an incidence of a security attack.

The remainder of this section provides a simplified description of each tactic in the reference taxonomy [55] illustrated in Figure 1.

#### 3.1.1. Detect Attacks

- *Detect Intrusion:* Comparing a system’s network traffic or service request patterns to a database of known malicious behavior signatures.
- *Detect Service Denial:* Comparing incoming network traffic to known *Denial of Service (DoS)* [80] attack profiles.
- *Verify Message Integrity:* Using checksums and hash values to ensure message and file integrity by using redundant information and unique strings.
- *Detect Message Delivery Anomalies:* Monitoring message delivery times and identifying abnormal connection patterns.

#### 3.1.2. Resist Attacks

- *Identify Actors:* Determining the source of any external input to the system; users are identified using user IDs, while other systems can be identified using access codes, IP addresses, protocols, ports, or other methods.
- *Authenticate actors:* Verifying an actor’s identity with passwords, one-time passwords, digital certificates, two-factor authentication, or biometric identification methods.
- *Authorize Actors:* Ensuring that an authenticated actor has the right to access and modify either data or services; this mechanism is usually enabled by providing access control mechanisms within a system.
- *Limit access:* Controlling access to computer resources by limiting the number of entry points and regulating the type of data allowed through.
- *Limit Exposure:* Minimizing damage caused by hostile actions by limiting data or services accessible through a single access point, thus reducing vulnerability to attacks.
- *Encrypt Data:* Encrypting to protect data and communication.
- *Separate Entities:* Entities can be physically separated on different servers, virtual machines, or an “air gap” with no electronic connection; additionally, sensitive data are kept separate from non-sensitive data, to reduce the risk of unauthorized access and attacks.
- *Validate Input:* Cleaning and checking input, using a security framework to filter, canonicalize, and sanitize input.
- *Change Credential Settings:* Change default security settings in systems and applications, to prevent unauthorized access; some systems may require users to change their passwords regularly for heightened security.

#### 3.1.3. React to Attacks

- *Revoke Access:* If an attack is suspected, access to sensitive resources may be restricted, even for legitimate users.
- *Restrict Login:* Repeated failed login attempts may indicate a potential attack, and access from a specific computer may be (perhaps temporarily) restricted.
- *Inform Actors:* If an attack is detected, its operators, personnel, or cooperating systems must be notified.

#### 3.1.4. Recover from Attacks

- *Audit:* Trace and identify attackers by analyzing audit trails.
- *Non-Repudiation:* Combining digital signatures and authentication by trusted third parties to prevent senders and recipients from denying message transmission and receipt, thus ensuring a secure and irrefutable record of communication.

### 3.2. Trade-Offs Among Security Tactics

Trade-offs are a key aspect of decision making as they help us recognize situations where a design choice prioritizes one quality attribute at the expense of another [70,81]. This is particularly beneficial for architects, as having a well-defined specification expands their range of options, which can be limited, especially for those with less experience [82].

Trade-offs usually center around quality attributes, so to assess their relevance, the non-functional requirements of the system being developed need to be outlined. Typically, comprehensive quality models like *ISO 25010* [36] provide an extensive classification of characteristics and subcharacteristics that a system may need to address its non-functional requirements. The applicability of this quality model to IoT is not well-documented. Some researchers have suggested specific IoT quality models based on the ISO 25010 standard [83,84,85], while others have identified common quality attributes of IoT systems [86,87,88,89], considering their diverse nature and resource constraints.

This research delves into identifying and defining specific trade-offs for IoT systems in line with the top-level quality attributes of *ISO 25010* [36], as outlined for each security tactic in the Bass et al. catalog [55]. Given this quality standard’s broad scope and coverage, it offers a flexible and comprehensive approach for IoT systems architects to have a catalog of enhanced tactics and understand their impact on quality attributes concerning various system and project requirements. Each tactic may impact one or more quality attributes, which can be recorded as ++ (make), + (help), − (hurt), or −− (break).

Table 2 details a qualitative trade-off analysis using this nomenclature [81]. If there is no significant impact, it is left blank. Recording the impacts allows us to analyze the trade-offs associated with each tactic. This means we can determine which specific quality attributes a tactic promotes and which ones it might compromise. This ability enables us to effectively compare different tactics when making decisions. Some quality attributes of *ISO 25010* [36], specifically *Functional Suitability*, *Compatibility*, and *Maintainability*, were excluded from consideration since the identified tactics do not influence these aspects.

Table 2 shows that security tactics affect quality attributes in IoT systems differently, reflecting necessary trade-offs between security and other aspects. While these tactics generally enhance reliability and security, certain ones, like *Detect Intrusion* and *Detect Message Delivery Anomalies*, negatively impact *Performance* due to their resource requirements for monitoring and attack detection.

Moreover, focusing heavily on *Security* can compromise *Interaction Capability*, which is problematic in IoT environments where *Interoperability* is essential. This trade-off analysis highlights the need to balance system protection with efficiency when choosing *Security* tactics.

The trade-offs associated with quality attributes for each tactic are outlined below.

#### 3.2.1. Detect Attacks

- *Detect Intrusion*: It hurts *Performance Efficiency* in IoT systems (- -), as it requires maintaining a permanent process of comparing malicious network traffic patterns and actions in the system with predefined signatures; this is demanding on memory and CPU. It favors *Reliability* (++) because it promotes prevention and early detection of attacks that can lead to availability problems or system failures. It favors *Safety* (++) because detecting intrusions in an IoT system is crucial to prevent them from harming the physical environment.
- *Detect Service Denial*: It hurts (- -) *Performance Efficiency* in IoT systems because of the continuous need to compare malicious network traffic patterns and system actions with predefined signatures, straining system resources. It favors (++) *Reliability* because it promotes prevention and early detection of attacks that can lead to availability problems or system failures. It favors *Flexibility* (++) by enabling early detection of networking bottlenecks that could hinder system scalability if not addressed on a timely basis. It favors *Safety* (++) because detecting *DoS* attacks in an IoT system is crucial for preventing interruptions to systems that monitor and act on critical infrastructure or people’s lives.
- *Verify Message Integrity*: It slightly hurts (—) *Performance Efficiency*, since generating and verifying hashes and checksums can impact an IoT system, especially if there is a high volume of messages, as this can be CPU-intensive. It favors (++) *Reliability*, as it prevents data manipulation by malicious users and avoids data integrity and consistency errors that could result in system failures. It favors (++) *Safety*, as it prevents data manipulation by malicious users attempting to execute specific commands in IoT systems operating as actuators.
- *Detect Message Delivery Anomalies*: It hurts (- -) *Performance Efficiency* because it requires constant monitoring of the messages exchanged by the IoT system, which uses additional system resources to analyze, process, and classify events that may be suspected of being an attack; it favors (++) *Reliability* because it promotes preventing and detecting attacks that can lead to availability problems or system failures; it favors (++) *Safety* since it prevents and mitigates the materialization of attacks that seek to inject commands or gain access to the IoT system to exploit functionalities for interaction with the environment.

#### 3.2.2. Resist Attacks

- *Identify Actors*: It favors (++) *Safety* by preventing and mitigating attacks aimed at injecting commands or gaining access to the IoT system for exploiting functionalities for interacting with the environment.
- *Authenticate Actors*: It could slightly hurt (—) *Interaction Capability* if many actions are required to complete a successful authentication. It favors (++) *Safety* by preventing and mitigating attacks aimed at injecting commands or gaining access to the IoT system for exploiting functionalities for interacting with the environment.
- *Authorize Actors*: It favors (++) *Safety* by preventing and mitigating attacks aimed at injecting commands or gaining access to the IoT system for exploiting functionalities for interacting with the environment.
- *Limit Access*: It favors (++) *Reliability* by preventing and detecting attacks that can cause availability issues or system failures. It favors(++) *Safety* by preventing and mitigating attacks that aim to inject commands or gain access to the IoT system to exploit functionalities for interacting with the environment.
- *Limit Exposure*: It favors (++) *Reliability* by proactively identifying and preventing attacks that may lead to availability problems or system crashes. It favors (++) *Safety* by thwarting and mitigating attacks designed to insert unauthorized commands or compromise IoT system access to exploit environmental interaction functionalities.
- *Encrypt Data*: It slightly hurts (—) *Performance Efficiency*, but some lightweight cryptographic algorithms can minimize the workload on the IoT system’s resources [90,91,92,93,94,95]. It favors (++) *Safety* by preventing and mitigating attacks that inject commands or gain access to the IoT system to exploit functionalities for interacting with the environment.
- *Separate Entities*: It favors (++) *Safety* by proactively preventing and countering potential attacks that seek to infiltrate the IoT system.
- *Validate Input*: It slightly hurts (—) *Performance Efficiency* if the validation logic is complex or requires external services. It favors (++) *Reliability* because it helps to prevent attacks that could lead to system failures resulting from the injection of control commands or other malicious parameter modifications. It favors (++) *Safety* by preventing attacks that can lead to system failures resulting from the injection of control commands or other parameter modifications for malicious purposes, which could dangerously impact the environment.
- *Change Credential Settings*: It favors (++) *Reliability* by preventing unauthorized third parties from accessing the IoT system with known default passwords and executing malicious actions. It favors (++) *Safety* by preventing attacks that can cause system failures due to injecting control commands or modifying parameters for malicious purposes, which could have a dangerous impact on the environment.

#### 3.2.3. React to Attacks

- *Revoke Access*: It favors (++) *Reliability* by containing the attack before it escalates. It favors (++) *Safety*, as it effectively can contain attacks aimed at injecting unauthorized commands or gaining unauthorized access to the IoT system, helping to safeguard the system and prevent the exploitation of its functionalities that could eventually impact the physical environment.
- *Restrict Login*: It may slightly hurt (—) *Interaction Capability* if a legitimate user is mistakenly blocked due to an error or enters their login credentials incorrectly, which could disrupt their access and hinder their ability to engage with the system or platform. It slightly favors (+) *Reliability* by preventing unauthorized users from accessing the IoT system and carrying out malicious actions. It favors (++) *Safety* by preventing and minimizing potential attacks that aim to inject unauthorized commands or gain unauthorized access to the IoT system, helping to safeguard the system’s functionalities and environmental interactions from exploitation.
- *Inform Actors*: It favors (++) *Reliability* because notifying actors about a potential attack helps ensure that actions are taken to contain and mitigate an attack. It favors (++) *Safety* because it prevents and mitigates attacks that seek to inject commands or gain access to the IoT system for environmental interaction.

#### 3.2.4. Recover from Attacks

- *Audit*: It slightly favors (+) *Reliability* by providing a detailed record of security actions or events, information that is crucial for conducting a root cause analysis related to specific incidents that impact the reliability of the IoT system. It favors (++) *Safety*, as it helps to prevent and mitigate attacks that attempt to inject commands or access the IoT system to exploit environmental interaction functionalities.
- *Non-repudiation*. It slightly hurts (—) *Performance Efficiency* because, although implementing non-repudiation may demand significant CPU and system resources, modern lightweight algorithms are available. It favors (++) *Safety* by preventing and mitigating attacks aimed at injecting commands or gaining unauthorized access to the IoT system to exploit environmental interaction functionalities.

## 4. Case Study: Nunatak—IoT Environmental Monitoring System

We illustrate the use of the trade-offs-aware secure IoT tactics with a real-world IoT Environmental Monitoring project, which integrates data from snow pollution sensors in remote mountain locations with a seaside academic laboratory. The system was developed for the Centre for Environmental Technologies (CETAM–UTFSM), a research center that studies the relationships between atmospheric chemical pollution and climate change.

### 4.1. Context

The average global temperature is expected to rise by several degrees Celsius this century, leading to natural disasters and significant social impacts. Climate change is primarily caused by increased human-made gases and aerosols intensifying the natural greenhouse effect [96]. Reducing short-lived climate pollutants (SLCPs) could slow down the retreat of glaciers and other ice-covered areas, known as the “cryosphere”. The Andes mountain range provides water for over 85 million people in 7 countries, and the impact of SLCPs has already been observed in this area. This presents an opportunity to study the transport of SLCPs to the Andean cryosphere and assess their impact on snow cover, water storage capacity, and chemical quality in mountainous areas [97].

**Figure 2:**
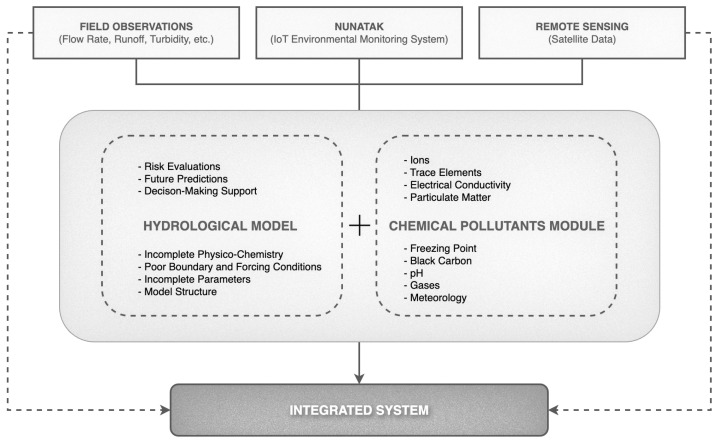
High-level operating model of the environmental integrated system.

This required to develop a hydrological model that combines information about chemical pollutants in the atmosphere and snow, high-resolution satellite images, advanced IoT remote sensing systems, and mathematical tools, to create an innovative integrated hydrological–chemical model. Figure 2 represents an overview of the hydro-chemical model and its main characteristics, where the IoT Environmental System serves as an input.

Figure 2 illustrates an integrated system for monitoring and modeling hydrological aspects and chemical contamination of the ecosystem. The system gathers data from three key sources: field observations (flow rate, runoff, and turbidity), the IoT monitoring system *NUNATAK*, and satellite remote sensing. Each source provides vital information for developing a hydrological model and a chemical contaminant module, aiding in risk assessments, future predictions, and decision support.

The hydrological model addresses incomplete data and model structure issues, while the chemical contaminant module analyzes ions, trace elements, electrical conductivity, pH, gases, and meteorological conditions. *NUNATAK* enhances the system by collecting real-time environmental data, improving the model’s accuracy and responsiveness. Integrating these data sources allows for a comprehensive understanding of the ecosystem, enabling informed decisions in environmental management.

### 4.2. Quality Attributes

The main non-functional requirements established as quality attributes were:

- *Confidentiality*: data in transit and at rest must be appropriately protected, to prevent unauthorized access by other systems or individuals, since the collected data are considered private and must be safeguarded and restricted from public access at all times.
- *Integrity*: unauthorized third parties must not be able to alter the stored or transmitted data, since any unauthorized modifications can lead to incomplete or inaccurate information, and significantly impact the descriptive and predictive findings from the hydrological–chemical model.
- *Availability*: the system must be available with a *Service Level Objective (SLO)* [98] of 99.9%; any incidents affecting availability can result in loss of collected data, negatively impacting the dataset used by the hydrological–chemical model, and service interruptions may require on-site problem resolution, necessitating travel to the remote experimental laboratory location.
- *Performance*: real-time information may be required, and any service degradation can affect telemetry data availability at a given time.

### 4.3. Threat Modeling and Mapping to Quality Attributes

We used security tactics to mitigate security threats, a concept well-documented in the literature [69,70]. Our first step was to identify and characterize the primary threats associated with the business problem. Even though there are catalogs of the most common IoT risks and vulnerabilities [99], there is not a widely accepted catalog to describes the main *threats* in IoT. But there are several threat models [10,100,101], so we identified threats using *STRIDE* [75,76], a methodology with a proven track record for identifying and modeling threats [70,102,103,104,105]. Table 3 outlines a threat identification that selects at least one threat for each *STRIDE* category (*Spoofing*, *Tampering*, *Repudiation*, *Information Disclosure*, *Denial of Service*, and *Elevation of Privilege*), ensuring comprehensive identification of threats in all aspects of the architectural problem.

Table 3 outlines identified threats labeled TH1 to TH5, each linked to one or more *STRIDE* categories. For instance, TH1 involves credential access and misuse, affecting *Spoofing (S)*, *Tampering (T)*, and *Repudiation (R)*. In contrast, TH5 represents a *Denial of Service (DoS)* attack, impacting *Availability (D)* and highlighting system vulnerabilities.

Table 4 correlates the threats in Table 3 with the case study security attributes, i.e., *Confidentiality*, *Integrity*, and *Availability (CIA)* (although *ISO 25010* [36] considers *Availability* as a subcharacteristic of *Reliability*).

Table 4 shows that the identified threats can impact multiple quality attributes, potentially compromising the security of the IoT system if they are not addressed. Additionally, this table acts as a traceability record, ensuring that threats have been identified for the various quality attributes of the system.

### 4.4. Illustrative Scenario: Man-in-the-Middle

To illustrate how the taxonomy of tactics can be applied, consider a scenario related to the *TH3* threat (see Table 3), which involves a *Man-in-the-Middle (MitM)* attack [106], which is a well-documented case in the context of IoT [107,108,109,110]. A *MitM* attack happens when an attacker covertly intercepts and potentially modifies the communication between two parties, such as a client and a server, without their knowledge; this allows the attacker to eavesdrop on confidential information and manipulate the data exchange without being detected, leading to a breach in the confidentiality and integrity of the system [107].

To evaluate the selection of tactics for this specific scenario, a team of three external experts defined the *Ground Truth* (i.e., correct answer) by characterizing tactics for a *MitM* scenario. Table 5 presents the experts’ demographics who created the *Ground Truth*.

Table 5 showcases the diverse experience of the study’s experts in IT consulting, insurance, telecommunications, and manufacturing. Their broad expertise in key security areas enhances the study’s robustness, bringing insights from professionals with extensive cybersecurity knowledge across various sectors.

The experts used a trade-offs-aware catalog of security tactics and held a work session to establish the ground truth through expert judgment, aiming to reach a consensus on the specified responses. The activities carried out in this session are specified below:

1. **Scenario Review**. The security experts analyzed the scenario regarding a *MitM* attack in an IoT environment.
2. **Tactics Selection**. Each expert independently chose security tactics from a catalog, considering system requirements and limitations.
3. **Justification**. Experts prepared brief reports justifying their selected tactics and addressing the scenario’s threats and trade-offs.
4. **Consensus Discussion**. They held a moderated discussion to compare selections, evaluate strengths, and strive for a consensus on the best tactics. After discussion, the experts refined their findings and chose the final tactics for the MitM scenario.

Table 6 presents the *Ground Truth* formulated by the experts.

Below is the rationale derived by experts to select tactics that resulted in the *Ground Truth* specified in Table 6.

- Detect Attacks
  - *Verify Message Integrity*: Verifying message integrity is an important aspect of design, as it ensures that the message sent is the same as the one received and has not been altered during transit through a communication channel. For instance, if a temperature sensor sends a message to a node or server, a hashing technique can be used to confirm that the message was not altered while in transit. Performance may be slightly compromised (—) when using this tactic.
- Resist Attacks
  - *Identify Actors*: Each IoT device must have a unique identity to establish traceability and subsequent authentication and authorization mechanisms. This identity can be represented by access codes, *IP* addresses, and other unique identifiers. Similarly, users who access these systems must have a unique and verifiable identity for authentication purposes. For instance, devices could utilize authentication tokens based on uniquely granted credentials. In a *MitM* scenario, it is essential to accurately identify all actors involved, including clients, servers, and devices. This is critical because an attacker could potentially impersonate any of these legitimate actors.
  - *Authenticate Actors*: IoT devices must undergo *mutual authentication* [41] with servers or other IoT devices to ensure secure communication. This entails both the IoT device and the server verifying each other’s identities before initiating communication. Protocols like *Transport Layer Security (TLS)* [112] can be utilized to achieve this *mutual authentication*. This approach prevents unauthorized communication interception, making it difficult for malicious actors to impersonate a legitimate device or deceive the server into accepting fraudulent connections. In addition, this method thwarts attackers from initiating unauthorized *MitM* connections, as they would also need to be authenticated. When authenticating people, it is best to do so transparently or automated to avoid negatively impacting negatively (—) *Interaction Capability*.
  - *Authorize Actors*: An IoT device must be restricted to perform only essential actions and access necessary resources upon successful authentication. An access control system enforces these restrictions, ensuring the IoT device can only execute specific actions. This system safeguards against attackers attempting to leverage an IoT device’s credentials to gain unauthorized access to resources or services.
  - *Encrypt Data*: Encryption is fundamental in preserving data integrity as it traverses between IoT devices and servers. End-to-end encryption is particularly significant in thwarting unauthorized access to sensitive information by ensuring that any intercepted messages remain incomprehensible without access to the encryption keys. In a *MitM* attack, encryption effectively upholds data confidentiality, even when transmitted across insecure networks. Since this tactic impacts performance only slightly negatively (—), it is important to use lightweight algorithms to mitigate the risk of significant impact on system performance.
- React to Attacks
  - *Inform Actors*: In a *MitM* attack, it is crucial to promptly notify IoT devices and system operators. IoT systems should have robust alert mechanisms to report suspicious activities. Real-time alerts should be established to notify IoT devices and administrators if an attacker attempts to disrupt communications, enabling them to take immediate action to prevent further damage.
- Recover from Attacks
  - *Audit*: Audit logs offer detailed insights into the success of the *MitM* attack. This helps address vulnerabilities and creates a historical record valuable for forensic investigations and preventing future attacks.

Some tactics were not used in solving this scenario because they were irrelevant or because selecting them would have meant significant trade-offs with key quality attributes. Specifically, the rationales for excluding these tactics are shown below.

- Detect Attacks
  - *Detect Intrusion*: A *MitM* attack can be seen as an intrusion in communication, but traditional intrusion detection methods may not be as effective in detecting *MitM* attacks since a successful attack can accurately replicate intercepted traffic; therefore, anomaly or signature detection techniques are not very helpful in detecting this type of attack.
  - *Detect Service Denial*: not applicable, as it is designed to detect *DoS* attacks.
  - *Detect Message Delivery Anomalies*: while this approach might offer insight into a potential *MitM* attack (it is not exclusive to this kind of attack), it significantly compromises the system’s performance (- -), which is a key project requirement; therefore, it was decided to employ alternative tactics rather than having a lesser impact on this aspect of system quality.
- Resist Attacks
  - *Limit Access*: restricting access does not prevent or defend against this kind of attack, since typically the attack occurs outside the systems, in the communication channels with other IoT devices or servers.
  - *Limit Exposure*: there is no direct intrusion into the system, since it involves intercepting communication channels.
  - *Separate Entities*: the *MitM* attack occurs outside the servers, and attempts to impersonate legitimate traffic.
  - *Validate Input*: a *MitM* attack does not rely on code injection or modification of any parameters that need validation.
  - *Change Credential Settings*: a *MitM* attack does not rely on system.
- React to Attacks
  - *Revoke Access*: revoking access for machine-to-machine authorization could disrupt the system’s operations, mainly since *MitM* attacks typically occur in communication channels rather than within the IoT system itself; for users who access IoT systems and fall victim to a *MitM* attack, blocking the user may be a better course of action.
  - *Restrict Login*: it does not mitigates *MitM* attacks.
- Recover from Attacks
  - *Non-repudiation*: This tactic could help against a *MitM* attack, by utilizing digital signatures to provide evidence of the sender’s and receiver’s identities; since in this scenario digital signatures are already used to verify message integrity, implementing this tactic is redundant, and would add unnecessary overhead, affecting the system performance (—).

## 5. Experimental Study

We conducted an experimental study to assess the impact of enhancing the security tactics taxonomy with IoT-specific trade-offs. The study was conducted in an industrial setting at Liricus SRL, a software company with over a decade of operations in Argentina and the USA. Twelve junior and senior practitioners worked on designing a secure IoT system using a catalog of architectural security tactics.

**Figure 3:**
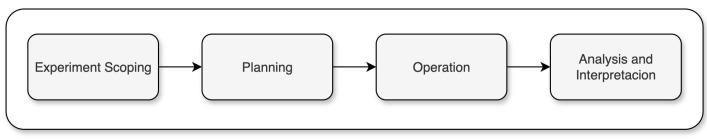
Experimental process followed in this study.

The study followed the method outlined by Wohlin at al. [113]; Figure 3 shows its stages.

As illustrated in Figure 3, the experimental method consists of several core stages. It begins with experiment scoping, which defines the problem statement, objectives, and goals for clarity. Next, the planning phase formulates the experimental design, instrumentation, hypotheses, and variables. The experiment is then operationalized according to this design, and data are systematically collected. Finally, the collected data are thoroughly analyzed and interpreted.

### 5.1. Experiment Scoping

The main objective of this activity is to assess the effectiveness of trade-off-aware IoT security tactics, for which the following goal definitions have been established.

- **Object of Study**: Effect and utility of the trade-offs-aware IoT security tactics catalog on practitioners’ design of secure IoT systems.
- **Purpose**: Assess the benefits for system designers of using a trade-offs-aware IoT security tactics catalog. While the existing catalog is established, it lacks trade-off evaluations and relevance to IoT system design. This study will analyze the impact of adding trade-off information by comparing practitioners’ decisions against a ground truth established by expert architects in a case study.
- **Perspective**: From the point of view of the researchers, determine if there are any consistent performance differences among individuals who chose specific tactics to design a secure IoT system that meets project requirements.
- **Quality focus**: Analyzing individual performance in selecting architectural tactics for a case study, assessing the subject’s effectiveness and efficiency, and the catalog’s usefulness in supporting architectural decision making.

### 5.2. Planning

This section describes in detail the design and planning of the experimental study.

#### 5.2.1. Hypothesis Formulation

We formulated several hypotheses (see Table 7) to assess how the IoT security tactics catalog helps practitioners to design secure IoT systems.

As shown in Table 7, the hypotheses are based on effectiveness, efficiency, utility, and accuracy in selecting architectural tactics. We assessed these parameters using performance metrics [114], which are commonly used in information retrieval [115] and machine learning [116] to evaluate algorithms in classification problems and decision making in binary-classification scenarios. They are also frequently used when individuals are making decisions within binary-classification problem-solving scenarios [117,118,119,120,121].

The metrics are:

- **Precision**. The proportion of correctly identified tactics (true positives) out of all tactics selected; a high precision score indicates that subjects are adept at selecting tactics with few false positives:
  (1) $$\mathrm{Precision}=\frac{TP}{TP+FP}$$
- **Recall**. The proportion of correctly identified tactics among all relevant tactics, as defined in the Ground Truth; a high recall score indicates that subjects can recognize tactics correctly with few false negatives:
  (2) $$\mathrm{Recall}=\frac{TP}{TP+FN}$$
- **F1-Score**. The harmonic mean of *Precision* and *Recall*, offering a unified metric that effectively balances both parameters:
  (3) $$\mathrm{Fl}-\mathrm{Score}=2\times \frac{\mathrm{Precision}\times \mathrm{Recall}}{\mathrm{Precision}+\mathrm{Recall}}$$
- **Accuracy**. The proportion of correctly identified tactics, including true positives and true negatives, out of the total tactics considered; this measure gives an overall performance assessment of the subject in correctly classifying positive and negative instances:
  (4) $$\mathrm{Accuracy}=\frac{TP+TN}{TP+TN+FP+FN}$$
  where
  - *TN* = tactics correctly NOT selected.
  - *TP* = tactics correctly selected.
  - *FN* = tactics that should be selected but were NOT selected.
  - *FP* = tactics that should NOT be selected but were selected.

#### 5.2.2. Variables Selection

Our experiment evaluates how practitioners select tactics from a standard catalog of security architectural tactics versus a trade-offs-aware version. We defined specific variables to measure participants’ performance in usefulness, effectiveness, efficiency, and accuracy. The key variables of the study are outlined below.

- Independent Variables
  - **Catalog**: Subjects receive one of the following two catalogs:
    * Standard catalog: A well-known catalog of security tactics [55].
    * Trade-offs-aware catalog: A catalog that includes the same tactics as the standard [55] but is enriched with trade-offs associated with each tactic and its impact on the quality attributes of the ISO 25010 standard [36] for the design of secure IoT systems.
- Dependent Variables. The dependent variables are the *Effectiveness*, *Efficacy*, *Usefulness*, and *Accuracy* of the selections, which are the focus of this study. We will analyze their correlation with performance metrics. Table 8 details the rationale for each metric assessing the *Efficiency*, *Effectiveness*, *Usefulness*, and *Accuracy* of the selections.

Table 8 outlines the alignment of performance metrics with hypothesis variables, essential for evaluating decision-making effectiveness in system design. Metrics like *Precision* and *Recall* assess efficiency and effectiveness. The F1-Score integrates these metrics to provide a comprehensive view of the tactics catalog’s usefulness. Additionally, *Accuracy* measures the correctness of designers’ selections, enhancing the reliability of the tactics selection process.

The case study scenario remains constant throughout the experiment and is consistent for all participants. As a result, it serves as a controlled factor to maintain consistency and ensures that any variations in the dependent variables are solely attributed to the type of catalog used.

#### 5.2.3. Experiment Design

The design involves one factor with two treatments: the factor is the tactics catalog, and the treatments are the standard catalog (control group, GR-1) and the trade-offs-aware catalog for designing secure IoT systems (experimental group, GR-2). Dependent variables range from 0 (no performance) to 1 (perfect performance).

We followed several key principles to add rigor and integrity of our experimental process [113]. A detailed overview of these principles is provided below.

- **Randomization**. We used a *stratified randomization* [113] approach to categorize twelve subjects into two groups, ensuring a mix of junior professionals (under five years of experience) and seniors (five years or more). Participants were randomly assigned to treatments, resulting in a slightly unequal distribution due to the odd number of seven seniors and five juniors, but we aimed for balance.
- **Blocking**. All participants received training before the experiment to ensure an equal understanding of tactics, eliminating disadvantages for those less familiar. Additionally, random group assignments helped minimize the effects of prior knowledge.
- **Balancing**. The experiment employed a balanced design with an equal number of subjects in each group to maintain fairness and reduce bias in findings.

Table 9 presents the list of participants in the experiment, their level of experience, the group to which they were assigned, and whether they had prior knowledge of tactics.

Table 9 shows that most participants reported no prior experience with tactics, except for one individual in GR-1 and two in GR-2. Although this difference could lead to variability in the approaches to tactic selection among the groups, measures were implemented to effectively address this potential issue and minimize its impact on the results.

#### 5.2.4. Instrumentation

The study included three phases: training, experimental, and post-experimental. In the training phase, participants were introduced to the study procedures. The experimental phase involved implementing interventions and collecting data. The post-experimental phase focused on follow-up assessments and data analysis. A survey was distributed before the first phase to collect information on participants’ seniority and experience with architectural tactics. Table 10 outlines the activities for each phase.

Table 10 outlines the study structure in three phases: training, experimental, and post-experimental. This design enables a systematic evaluation of how provided materials affect participants’ performance. In the experimental phase, different tactics catalogs were used to analyze the impact of the IoT-enhanced catalog on security tactics selection compared to the standard version. The post-experimental phase gathered participant feedback, offering insights into the usability and perception of the tactics and emphasizing the benefits of IoT-specific enhancements in security decision making.

### 5.3. Operation

This phase involves three steps: *preparation*, involving subject selection and form preparation; *execution*, where subjects perform tasks under different treatments and data are collected; and *data validation*, where collected data are verified.

- **Preparation**. Subjects were unaware of the specific aspects under study to eliminate bias. They were informed that the research focused on the practicality of tactics catalogs, not on specific hypotheses. All necessary materials were provided, and anonymity was assured to promote honest participation.
- **Execution**. The study took place during a 90-min session on a regular workday, with data collected primarily through online forms. A brainstorming session at the end gathered feedback on the experiment’s design and dynamics.
- **Data Validation**. Tactics were chosen using closed-end forms via *Google Forms* (https://workspace.google.com/products/forms/ (accessed on 10 November 2024)). This approach limited responses to a pre-defined list, ensuring only cataloged elements were included. Completeness of responses was reviewed to verify adherence to instructions.

### 5.4. Analysis and Interpretation

To analyze the collected data, we will compare the experimental data with the expert-derived *Ground Truth* and evaluate how each subject’s responses affect performance metrics. Next, we will use descriptive statistics to assess central tendency and dispersion. Lastly, we will conduct hypothesis testing and a *bootstrap* analysis [122] to enhance our findings.

#### 5.4.1. Matching the *Ground Truth*

We used a *loss function* [123] to quantify the cost associated with an event or variable values [116]; this function gives “1” to an incorrectly classified tactic (as per the Ground Truth), and “0” to a correctly classified tactic. The *average loss function* is:

(5) $$L_{01}=\frac{1}{n}\sum_{i=1}^{n}L_{\left(\hat{y_{i}},y_{i}\right)}\;\mathrm{with}\;L_{\left(\hat{y_{i}},y_{i}\right)}=\left\{\begin{array}{cc} 0, & \mathrm{if}\;{\hat{y}}_{i}=y_{i}\\ 1, & \mathrm{if}\;{\hat{y}}_{i}\neq y_{i} \end{array}\right.$$

where

- ${\hat{y}}_{i}$ = the selection or omission of a particular tactic specified in the *Ground Truth*;
- $y_{i}$ = the selection or omission of a particular tactic performed by the subjects;
- $L_{(\hat{y_{i}},y_{i})}$ = a *loss function* that assigns a value of “0” or “1” based on the match with the *Ground Truth*;
- *n* = total number of tactics in the catalog.

Table 11 shows the *loss function* and the *Ground Truth* for each subject. In the *GT (Ground Truth)* column, a “0” indicates that this tactic was selected, and a “1” indicates that it was not selected. For the remaining columns (S1 to S12), a “0” indicates a subject’s response matching the *Ground Truth*, and a “1” indicates a mismatch.

Based on the data in Table 11, the tactics *Limit Access*, *Change Credential Settings*, and *Restrict Login* had the highest error rates, each with seven incorrect selections. In *Limit Access*, errors were split between four in GR-1 and three in GR-2. *Change Credential Settings* had five errors in GR-2 and two in GR-1, while *Restrict Login* recorded three errors in GR-1 and four in GR-2. The least erroneously selected tactics were *Detect Service Denial* and *Detect Message Delivery Anomalies*, each with one error.

Average losses indicated that GR-2 outperformed GR-1 (0.3 vs. 0.36). For juniors, GR-1 was 0.33 compared to 0.28 in GR-2, while seniors had higher losses (GR-1 at 0.39 and GR-2 at 0.31), suggesting that more experience did not improve their effectiveness in applying IoT security tactics.

#### 5.4.2. Descriptive Statistics

To enhance the statistical analysis of our experiment, we will calculate central tendency measures such as the *Mean*, *Median*, and *Standard Deviation (SD)* applied to the performance metrics. Table 12 shows a statistical analysis of the performance metric results, at both group level and within clusters.

Table 12 shows that GR-2 outperforms GR-1 across all metrics, with higher mean and median values. This supports the idea that a trade-off enriched catalog enhances participants’ decision making in IoT contexts. GR-2 also exhibits lower variability, indicating more consistent results. Notably, junior participants in both groups often match or exceed senior performance, especially in GR-2, where juniors significantly outshine those in GR-1. This suggests that a tailored catalog of tactics empowers juniors to make better decisions without relying solely on experience.

**Figure 4:**
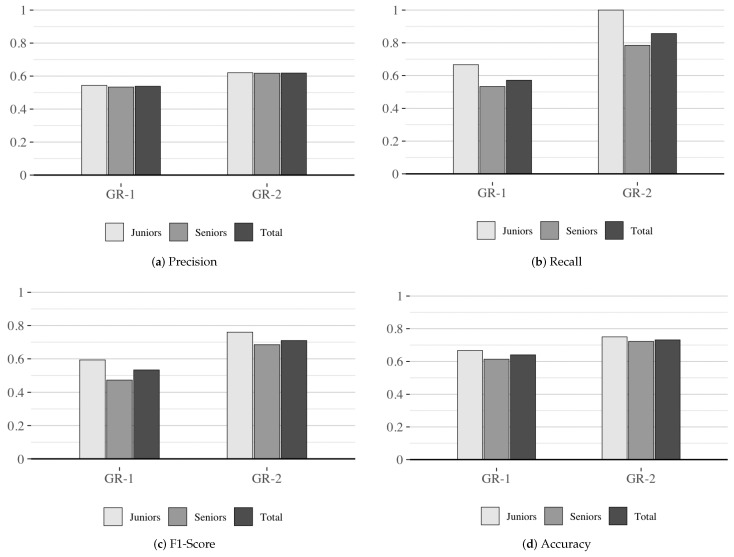
Average performance metrics for each experimental group and clusters.

Figure 4 illustrates the average performance metrics for each group, showing that GR-2 outperforms GR-1 across all metrics, irrespective of seniority level. The most significant disparity is evident in the *Recall* and *F1-Score* metrics. Additionally, a consistent pattern emerges where the junior cluster consistently achieves equal or superior results compared to the senior cluster in GR-1 and GR-2 across all average metrics.

#### 5.4.3. Hypothesis Testing

Due to our experiment’s small sample size, methodological decisions were made based on established literature to ensure the validity and reliability of the results. These decisions, rooted in recommended practices for studies with small samples, were implemented to bolster the robustness of the statistical analysis.

To compare the experimental groups, we employed the non-parametric *Mann–Whitney* test [113] to avoid assuming the normality of the data [113,124]. A significance level of α = 0.1 instead of the conventional level of 0.05 [125,126,127] was set due to the limited sample size, in accordance with studies that suggest this adjustment to improve the detection of significant effects in similar contexts [113,124,128]. Table 13 describes the results of hypothesis testing using the *Mann–Whitney* test of our experiment’s working hypotheses.

As shown in Table 13, for *Precision*, a *U statistic* of 9.0 and a *p*-value of 0.16 indicate that the null hypothesis ($H_{00}$) cannot be rejected, with a limited effect size of 0.24. For *Recall*, the *U statistic* was 6.5 with a *p*-value of 0.07, allowing us to reject the null hypothesis ($H_{10}$) in favor of the alternative, supported by a moderate effect size of 0.30. The *F1-Score* showed significant results as well, with a *U statistic* of 7.0 and a *p*-value of 0.08, leading to the rejection of the null hypothesis ($H_{20}$) and an effect size of 0.29. For *Accuracy*, a *U statistic* of 9.5 and a *p*-value of 0.18 indicate that the null hypothesis ($H_{30}$) is not rejected, with an effect size of 0.22 showing no significant impact from the enriched catalog.

#### 5.4.4. Bootstrapping the Sample

We applied the *bootstrap* technique with 1000 iterations to evaluate performance differences between GR-1 and GR-2, following literature recommendations for accurate confidence intervals [129,130]. This method involved resampling with replacement, and we assessed performance using the *medians* for *Precision*, *Recall*, *F1-Score*, and *Accuracy*, chosen for their robustness to outliers [124]. We used 90% confidence intervals to better capture subtle data variations while maintaining rigor in studies with small samples. Table 14 displays the confidence intervals for 250, 500, 750, and 1000 iterations, highlighting their relevance in our analysis.

As shown in Table 14, metrics for *Precision*, *Recall*, *F1-Score*, and *Accuracy* stabilized from 750 iterations onward, indicating consistent performance. Thus, the results at 1000 iterations align with best practices, suggesting stable convergence and minimal potential for improvement. At 1000 iterations, *Precision* showed no significant differences between groups (CI: [−0.06, 0.23]), suggesting the enriched catalog did not affect precision. However, *Recall* significantly improved (CI: [0.07, 0.57]), indicating enhanced identification of relevant tactics. The *F1-Score* also differed significantly (CI: [0.03, 0.38]), reflecting a better balance between *Precision* and *Recall*. In contrast, *Accuracy* showed no significant differences (CI: [0, 0.24]).

## 6. Discussion

The experimental results evidence that incorporating IoT-specific trade-offs in the enhanced catalog empowers designers to make more informed decisions than traditional catalogs. This study’s significant contribution demonstrates how trade-offs facilitate decision making by balancing quality attributes like performance, security, and reliability, which are crucial in IoT environments. The stability observed in the performance metrics of the experimental group supports the hypothesis that the enhanced catalog not only improves the tactic selection but also reduces variability in decisions, particularly among less experienced professionals.

An important finding is that GR-2 maintained superior performance in all metrics, regardless of the subjects’ experience level. This suggests that knowledge presented as trade-offs helps experienced professionals refine their decisions and enables less experienced individuals to make decisions comparable to senior practitioners. This highlights the utility of the enhanced catalog as a support tool for designers of secure IoT systems, irrespective of their experience level. Furthermore, junior participants either matched or outperformed senior participants in all performance metrics in both groups, with a more pronounced effect in certain metrics of GR-2, such as *Recall*, where juniors achieved the maximum score (1), 21 points higher than seniors (0.79) in both the *mean* and *median* (see Table 12). This could be attributed to the preference of senior professionals to rely on their knowledge rather than catalogs.

Although the enriched catalog was helpful, some tactics require further clarification as they were selected erroneously. These tactics include *Limit Access*, *Change Credential Settings*, and *Restrict Login* because their selection errors were observed in both groups (see Table 11). This could be attributed to the need for improved description in the catalog or indicate a common issue in the design strategy for optimal tactic selection because while trade-offs with quality attributes of *ISO 25010* [36] significantly enhance the catalog’s usefulness, the strategies for selecting an optimal number of tactics for a specific case are not explored. The subjects’ diverse selection strategies range from conservative approaches that select the minimum number of tactics to riskier ones that choose tactics not strictly required. Correct tactic selection significantly impacts the implementation effort, design and development time, and, ultimately, the project budget.

We validated two working hypotheses ($H_{11}$ and $H_{21}$) (see Table 7), showing that the trade-offs-aware catalog enhances the effectiveness and usefulness of decisions made by secure IoT system designers. However, there was no significant difference between the groups to validate hypotheses $H_{01}$ and $H_{31}$. This raises an interesting point for analysis regarding *Precision* and *Accuracy* metrics related to these hypotheses. The high *Recall* results of the experimental group (*mean* = 0.86 and *median* = 0.93) could potentially impact *Precision* due to increased tolerance for false positives. Additionally, the *F1-Score*, which balances *Precision* and *Recall*, supports rejecting hypothesis $H_{20}$ in favor of $H_{21}$ (see Table 7). Concerning *Accuracy*, the sample size could influence the number of false positives and negatives, impacting this metric. For instance, if one or two subjects make mistakes in selecting tactics, it can greatly decrease the overall accuracy (see Equation (4)), leading to a distorted interpretation of the catalog’s performance.

Despite employing a thorough validation approach and implementing well-established measures for dealing with small sample sizes, such as utilizing the *Mann–Whitney* test, relaxing the significance level, conducting effect size analysis, and employing *bootstrap* methods, it is essential to acknowledge the constraints imposed by the limited sample size. While the results are promising and exhibit a consistent trend, it is possible that certain effects may not have been fully evident due to the small participant pool, and their generalization should be approached with caution. Hence, future studies will corroborate the findings and explore how the enhanced catalog impacts more diverse IoT systems with varied characteristics.

The experimental results were complemented by a case study analysis demonstrating the application of a security tactics taxonomy in a real-world IoT Environmental Monitoring System. This validated the proposed taxonomy’s practical applicability, evidencing that the enriched catalog’s contributions have a tangible impact on implementing secure IoT systems rather than being purely theoretical. However, it would be interesting to explore how this catalog performs in other IoT domains, such as healthcare or smart cities, where trade-offs may have different impacts, especially given the increasing concern regarding IoT cybersecurity.

## 7. Threat to Validity

To evaluate the study’s validity, we identified and addressed several potential threats, categorizing them into four main areas: internal validity, external validity, construct validity, and conclusion validity [131].

- Internal Validity: A potential threat to validity is learning bias, where participants in the experimental group may have improved their performance due to exposure to additional material provided by the IoT trade-offs-aware catalog. We ensured each group received the specific catalog during the experiment to address this. Another potential threat to the internal validity of this experiment is that some participants may have prior knowledge of the security tactics. To address this concern, we conducted a training session before the experiment in which all the tactics in the catalog were presented and explained to both the control and experimental groups. This approach ensures that all participants, regardless of their previous experience, have a uniform understanding of the tactics. As a result, we eliminate any potential bias arising from prior knowledge and establish equivalent initial conditions for both groups. Additionally, we utilized *stratified random* assignment [113] to achieve a balanced representation of junior and senior participants in both groups. Another potential threat to the internal validity is bias from the experts who established the ground truth. To address this, we selected three external experts to incorporate diverse perspectives and minimize individual biases. They provided objective assessments since they were not involved in the study’s outcomes. Discussion sessions were held to compare their selections and reach a consensus, ensuring the chosen tactics were based on a thorough evaluation of the associated threats and limitations.
- External Validity: A potential concern is using hypothetical scenarios that provide conceptual clarity but may not capture the real-world complexities, constraints, and considerations in designing secure IoT systems. We addressed this concern by using a real-world case and conducting threat modeling using *STRIDE* [75] to accurately identify security threats that could be addressed using specific tactics [70]. Furthermore, the study was conducted in an industrial setting with practitioners of varying experience levels, which is a common scenario in the industry and supports the potential for replicating the study accurately. Additionally, all the necessary information to reproduce and validate the study’s results, including the dataset containing detailed performance metric results from our experiment, is publicly available (https://doi.org/10.5281/zenodo.13896006).
- Construct Validity: We used commonly accepted performance metrics to measure the fitness of tactic selection. These metrics are standard in performance evaluation and have been previously used in several studies [117,118,119,120,121]. The *Precision*, *Recall*, *F1-Score*, and *Accuracy* metrics were linked to key decision-making concepts such as efficiency, effectiveness, utility, and accuracy, respectively, to give practical meaning to the hypotheses (see Table 8). To prevent bias of the subjects, details of the hypothesis or aspects that could influence the subjects’ behavior and impact the results were not disclosed. To avoid bias in the *Ground Truth* (see Table 6), the analysis was conducted by a group of three external experts (see Table 5) using a consensus-based approach to determine the appropriate set of tactics for the case study scenario. This ensured that standards of integrity and objectivity were maintained in the evaluation.
- Conclusions Validity: The small sample size limits generalizing the results and reduces the statistical power. While finding large samples for experimental studies in industrial contexts is a well-known problem [113], there are techniques to address this issue [113,124,125,126,127,128]. In our case, we relaxed the statistical significance (α = 0.1), used *bootstrap* simulations with 1000 iterations, and conducted hypothesis tests and effect size analysis using the *Mann–Whitney* non-parametric test, which has proven helpful with small samples [113]. Even though we carefully followed the literature’s recommendations for small samples, we believe that future study replications with a larger sample would yield more representative results. However, the obtained results, particularly in the *Recall* and *F1-Score* metrics, demonstrate a clear and consistent trend, highlighting the practical usefulness of the enriched catalog with trade-offs for designing secure IoT systems.

## 8. Conclusions

This work explores how enhancing a general-purpose security tactics catalog with trade-offs related to quality attributes among *ISO 25010* [36] can help practitioners design secure IoT systems. To validate the proposal, two approaches were used: (1) documenting a real-world case study involving an IoT Environmental Monitoring System to demonstrate the applicability of security tactics and their trade-offs concerning project requirements and (2) conducting an experimental study involving twelve practitioners from a software factory to validate the efficiency, effectiveness, usefulness, and accuracy of the decisions made by designers with the IoT trade-offs-aware catalog.

To address the practical case scenario, the experimental setup involved two groups: a control group (GR-1) using the standard catalog and an experimental group (GR-2) using the enriched catalog. Four alternative hypotheses were established to validate the efficiency, effectiveness, usefulness, and accuracy of the designers’ decisions in each group. We used well-known metrics such as *Precision*, *Recall*, *F1-Score*, and *Accuracy* to measure the subjects’ performance. The experiment results indicated that the average values of all performance metrics were higher in GR-2 than in GR-1. However, only some metrics were statistically significant, specifically *Recall* and *F1-Score*, leading to the rejection of the null hypotheses $H_{10}$ and $H_{20}$ (see Table 13), respectively. Due to the sample size, the analysis was complemented with other formal techniques suitable for working with small samples, such as *bootstrap*, to reinforce the results obtained in the previous analyses.

The research revealed variability in tactic selection, with some participants opting for fewer tactics while others selected many, which decreased precision. Junior participants outperformed seniors, indicating that structured guidance may benefit less experienced designers in tackling IoT security challenges.

The key contributions are: (1) an approach to build scenario-specific trade-offs-aware architecture guidelines upon general purpose, attribute-specific design knowledge; (2) an IoT-specific trade-offs-aware taxonomy of architectural tactics to support design of secure IoT systems; and (3) empirical evidence that an IoT-specific trade-offs-aware taxonomy of architectural tactics offers effective, useful guidance for designing secure IoT systems.

While this study employed a thorough experimental validation methodology, it is important to acknowledge its limitations, particularly the small size of the experimental sample. Future investigations should aim to validate this approach using a larger dataset. Additionally, it would be beneficial to explore this taxonomy across various IoT domains, where the trade-offs might differ based on the specific requirements of each sector.

As ongoing work, we are developing a recommender system using *Large Language Models (LLM)* [132,133] to assist in the design of secure IoT systems, which is a hot topic in the software architecture community [134,135,136,137]. In future work, we plan to replicate the experiment in both industrial and academic contexts within IoT domains such as healthcare, smart cities, and manufacturing to assess the influence of the choice of security tactics in designing secure IoT systems.

## Figures and Tables

**Table 1:** Summary of approaches for designing secure IoT systems.

Reference	Type	Contributions	Limitations
Fernández (2011) [41]	Patterns	Catalog of security patterns	Not specific for IoT, Trade-offs not addressed
Fernández (2020) [44]	Patterns	Pattern for a Secure IoT Architecture	Trade-offs not addressed
Fernández et al. (2020) [45]	Patterns	Secure Publish/Subscribe pattern for IoT	Trade-offs not addressed
Fernández et al. (2022) [47]	Patterns	Secure IoT Thing design pattern	Trade-offs not addressed
Fernández et al. (2022) [49]	Patterns	Abstract Security Patterns	Not specific for IoT, Trade-offs not addressed
Orellana et al. (2019) [50]	Patterns	Taxonomy for Security Patterns	Trade-offs addressed only for case study
Orellana et al. (2022) [1]	Patterns	Pattern for Secure Sensor Node	Trade-offs not addressed
Orellana et al. (2022) [51]	Patterns	Pattern for Secure Actuator Node	Trade-offs not addressed
Schumacher et al. (2013) [42]	Patterns	Security Patterns for IT systems	Not specific for IoT, Trade-offs not addressed
Bass et al. (2021) [55]	Tactics	Taxonomy for Security Tactics	Not specific for IoT, Trade-offs not addressed
Colesky et al. (2016) [67]	Tactics	Taxonomy for Privacy	Not specific for IoT, Trade-offs not addressed, Privacy-centric
Erder et al. (2021) [62]	Tactics	Taxonomy for Security Tactics	Not specific for IoT, Trade-offs not addressed
Fernández et al. (2015) [65]	Tactics	Taxonomy for Security Tactics	Not specific for IoT, Trade-offs not addressed
Rozanski and Woods (2011) [60]	Tactics	Taxonomy for Security Tactics	Not specific for IoT, Trade-offs not addressed
Ryoo et al. (2012) [66]	Tactics	Taxonomy for Security Tactics	Not specific for IoT, Trade-offs not addressed
Bashir et al. (2022) [73]	Reference Architectures	Reference architecture for IoT smart buildings	Domain-specific, Not security-focused, Trade-offs not addressed
ISO/IEC 30141 (2024) [34]	Reference Architectures	Reference Architecture for IoT	Not security-focused, Insufficient Design Guidance, Trade-offs not addressed
Szmeja et al. (2023) [74]	Reference Architectures	Reference Architecture for Next Generation IoT (NGIoT)	Not security-focused, Trade-offs not addressed
Syed et al. (2018) [79]	Threat Modeling	A Misuse Pattern for DDoS in IoT	Focus on misuse, Trade-offs not addressed

**Table 2:** Trade-offs among security tactics.

Security Tactic	Quality Attribute					
	Performance Efficiency	Interaction Capability	Reliability	Security	Flexibility	Safety
**Detect Attacks**						
Detect Intrusion	- -		++	++		++
Detect Service Denial	- -		++	++	++	++
Verify Message Integrity	—		++	++		
Detect Message Delivery Anomalies	- -		++	++		++
**Resist Attacks**						
Identify Actors				++		++
Authenticate Actors		—		++		++
Authorize Actors				++		++
Limit Access			++	++		++
Limit Exposure			++	++		++
Encrypt Data	—			++		++
Separate Entities				++		++
Validate Input	—		++	++		++
Change Credential Settings			++	++		++
**React to Attacks**						
Revoke Access			++	++		++
Restrict Login		—	+	++		++
Inform Actors			++	++		++
**Recover from Attacks**						
Audit			++	++		++
Non-repudiation	—			++		++

**Table 3:** Threat classification following the *STRIDE* modeling methodology.

Threat ID	Description	S	T	R	I	D	E
TH1	Gaining access to and misusing credentials that were originally granted to someone else	✓	✓		✓		
TH2	Attempting to gain unauthorized system access with a brute force attack, i.e., systematically trying combinations of usernames and passwords	✓	✓		✓		
TH3	Performing a *Man-in-the-Middle (MitM)* attack to intercept, manipulate, or eavesdrop on the data transmitted to the IoT Environmental System	✓	✓		✓		
TH4	Gaining system access by exploiting a previously unidentified or unaddressed software vulnerability, allowing them to read and alter data	✓	✓	✓	✓		✓
TH5	Performing a *Denial of Service (DoS)* attack to make the IoT Environmental System unavailable and non-functional					✓	

**Table 4:** Mapping security threats with quality attributes.

Threat ID	Quality Attribute		
	**Confidentiality**	**Integrity**	**Availability**
TH1	✓	✓	
TH2	✓	✓	
TH3	✓	✓	
TH4	✓	✓	
TH5			✓

**Table 5:** Experts involved in the study, their years of experience, industries they’ve been involved in, and their expertise in *Certified Information Systems Security Professional (CISSP)* security domains [111].

ID	Experience (Years)	Industry	Security Domain
E1	19	IT Consulting, Insurance, Health, Sport, Wellbeing and Fitness; Telecommunications, R&D	Security and Risk Management; Asset Security, Security Architecture and Engineering, Communication and Network Security, Identity and Access Management (IAM), Security Assessment and Testing, Security Operations, Software Development Security
E2	16	IT Consulting, Insurance, Sport, Wellbeing and Fitness; Telecommunications, Natural Resources	Security Architecture and Engineering, Communication and Network Security, Identity and Access Management (IAM), Security Assessment and Testing, Security Operations, Software Development Security
E3	15	IT Consulting, IT Services, Insurance, Health, Wellbeing and Fitness; Flavors and Fragrances Manufacturing Telecommunications, Natural Resources, Consumer Electronics Manufacturing and Retail	Security and Risk Management; Asset Security, Security Architecture and Engineering, Communication and Network Security, Identity and Access Management (IAM), Security Operations, Software Development Security

**Table 6:** Ground Truth of correct answers for the *MitM* scenario prepared by experts.

Category	Tactic ID	Tactic Name	Selected
**Detect Attacks**	TA1	Detect Intrusion	
	TA2	Detect Service Denial	
	TA3	Verify Message Integrity	✓
	TA4	Detect Message Delivery Anomalies	
**Resist Attacks**	TB1	Identify Actors	✓
	TB2	Authenticate Actors	✓
	TB3	Authorize Actors	✓
	TB4	Limit Access	
	TB5	Limit Exposure	
	TB6	Encrypt Data	✓
	TB7	Separate Entities	
	TB8	Validate Input	
	TB9	Change Credential Settings	
**React from Attacks**	TC1	Revoke Access	
	TC2	Restrict Login	
	TC3	Inform Actors	✓
**Recover from Attacks**	TD1	Audit	✓
	TD2	Non-repudiation	

**Table 7:** Experimental study hypotheses.

Null Hypothesis	Alternative Hypothesis
$H_{00}$: Enriching the security tactics catalog with trade-offs for designing secure IoT systems does not improve practitioners’ architectural design decisions’ efficiency	$H_{01}$: The trade-offs-aware catalog offers enhancements over the standard catalog, boosting the overall efficiency of the decisions made by secure IoT system designers
$H_{10}$: Enriching the security tactics catalog with trade-offs for designing secure IoT systems does not improve practitioners’ architectural design decisions’ effectiveness	$H_{11}$: The trade-offs-aware catalog offers enhancements over the standard catalog, boosting the overall effectiveness of the decisions made by secure IoT system designers
$H_{20}$: Enriching the security tactics catalog with trade-offs for designing secure IoT systems does not improve practitioners’ architectural design decisions’ usefulness	$H_{21}$: The trade-offs-aware catalog offers enhancements over the standard catalog, boosting the overall usefulness of the decisions made by secure IoT system designers
$H_{30}$: Enriching the security tactics catalog with trade-offs for designing secure IoT systems does not improve practitioners’ architectural design decisions’ accuracy	$H_{31}$: The trade-offs-aware catalog offers enhancements over the standard catalog, boosting the overall accuracy of the decisions made by secure IoT system designers

**Table 8:** The reasoning for aligning performance metrics with hypotheses variables.

Variable	Metric	Rationale
**Efficiency**	Precision	It refers to the designers’ ability to choose the maximum number of correct tactics while minimizing the selection of irrelevant tactics.
**Effectiveness**	Recall	It refers to the ability of designers to successfully address and resolve problems by utilizing appropriate tactics. When the individuals choose the most relevant tactics available, they can effectively resolve the practical case scenario.
**Usefulness**	F1-Score	It refers to the practical value of tactics for designers. It encompasses aspects of effectiveness and efficiency, considering how valuable the catalog is for practical decision making. A high F1-Score indicates high usefulness, as it enables designers to be both effective and efficient.
**Accuracy**	Accuracy	It measures how many selections designers made were correct, whether positive or negative. It can help to provide an overall view of the performance of the tactics selection process.

**Table 9:** Subjects participating in the study according to their experience and group assignment.

Subject ID	Experience (Years)	Group	Prior Knowledge of Architectural Tactics
**S1**	1	GR-1	No
**S2**	3	GR-1	No
**S3**	15	GR-1	No
**S4**	2	GR-1	No
**S5**	10	GR-1	No
**S6**	15	GR-1	Yes
**S7**	5	GR-2	No
**S8**	15	GR-2	No
**S9**	1	GR-2	No
**S10**	17	GR-2	No
**S11**	2	GR-2	Yes
**S12**	16	GR-2	Yes

**Table 10:** Activities carried out in the context of the experimental study phases.

Phase	Duration (minutes)	Activities
**Training**	20	- Presentation of the study - Introduction on Architectural Tactics
**Experimental**	60	- Presentation of the case study scenario - Group division: participants were divided into control and experimental groups - Documentation: GR-1 received a catalog with security tactics and the case study scenario; GR-2 received an enhanced catalog of IoT security tactics and the case study scenario - Scenario-based decision making
**Post-Experimental**	10	- Gathering feedback from the subjects

**Table 11:** Assessing the subjects responses with the *Ground Truth* using a *loss function*.

MitM Scenario													
		**GR-1**						**GR-2**					
**Security Tactic**	**GT**	S1	S2	S3	S4	S5	S6	S7	S8	S9	S10	S11	S12
**Detect Attacks**													
Detect Intrusion	**0**	1	1	0	1	1	0	0	0	0	0	0	0
Detect Service Denial	**0**	0	0	0	0	1	0	0	0	0	0	0	0
Verify Message Integrity	**1**	1	0	0	0	1	0	1	0	0	0	0	0
Detect Message Delivery Anomalies	**0**	0	0	0	0	0	0	0	0	0	0	0	1
**Resist Attacks**													
Identify Actors	**1**	1	1	1	0	0	1	0	1	0	1	0	0
Authenticate Actors	**1**	0	1	1	0	0	0	0	0	0	0	0	0
Authorize Actors	**1**	0	1	1	0	0	0	0	0	0	1	0	0
Limit Access	**0**	1	0	0	1	1	1	1	0	1	1	0	0
Limit Exposure	**0**	0	0	1	0	0	1	1	0	1	0	0	0
Encrypt Data	**1**	0	0	0	0	0	1	1	0	0	0	0	0
Separate Entities	**0**	0	1	0	0	0	1	0	0	0	0	0	0
Validate Input	**0**	0	0	0	0	1	0	0	1	1	0	0	0
Change Credential Settings	**0**	1	1	0	0	0	0	1	0	1	1	1	1
**React to Attacks**													
Revoke Access	**0**	0	0	0	1	1	0	1	1	1	1	0	0
Restrict Login	**0**	1	1	0	0	0	1	1	0	1	1	1	0
Inform Actors	**1**	0	1	1	0	1	1	0	0	0	1	0	0
**Recover from Attacks**													
Audit	**1**	0	0	1	0	0	1	0	0	0	0	0	0
Non-repudiation	**0**	1	0	0	0	0	0	1	1	1	0	1	1
**∑**		7	8	6	3	7	8	8	4	7	7	3	3
** $L_{01}$ **		0.39	0.44	0.33	0.17	0.39	0.44	0.44	0.22	0.39	0.39	0.17	0.17
Avg. $L_{01}$ (juniors)		0.33						0.28					
Avg. $L_{01}$ (seniors)		0.39						0.31					
Total Avg. $L_{01}$		0.36						0.3					

**Table 12:** Measures of the central tendency of performance metrics for each group and cluster.

Metric	Group	Cluster	Mean	Median	Std. Dev.
**Precision**	GR-1	Juniors	0.54	0.5	0.14
		Seniors	0.53	0.5	0.12
		**Total**	**0.54**	**0.5**	**0.12**
	GR-2	Juniors	0.62	0.62	0.11
		Seniors	0.62	0.64	0.14
		**Total**	**0.62**	**0.64**	**0.12**
**Recall**	GR-1	Juniors	0.67	0.57	0.3
		Seniors	0.48	0.43	0.21
		**Total**	**0.57**	**0.5**	**0.25**
	GR-2	Juniors	1	1	0
		Seniors	0.79	0.79	0.19
		**Total**	**0.86**	**0.93**	**0.18**
**F1-Score**	GR-1	Juniors	0.59	0.53	0.2
		Seniors	0.47	0.43	0.1
		**Total**	**0.53**	**0.48**	**0.16**
	GR-2	Juniors	0.76	0.76	0.08
		Seniors	0.69	0.69	0.14
		**Total**	**0.71**	**0.75**	**0.12**
**Accuracy**	GR-1	Juniors	0.67	0.61	0.14
		Seniors	0.61	0.61	0.06
		**Total**	**0.64**	**0.61**	**0.1**
	GR-2	Juniors	0.75	0.75	0.11
		Seniors	0.72	0.75	0.13
		**Total**	**0.73**	**0.75**	**0.11**

**Table 13:** Hypothesis testing using *Mann–Whitney* test.

Hypothesis	Metric	Statistic (U)	*p*-Value	Significance (α = 0.1)	Effect Size (r)
$H_{00}$, $H_{01}$	**Precision**	9.0	0.16	Non-Significant	0.24
$H_{10}$, $H_{11}$	**Recall**	6.5	0.07	Significant	0.30
$H_{20}$, $H_{21}$	**F1-Score**	7.0	0.08	Significant	0.29
$H_{30}$, $H_{31}$	**Accuracy**	9.5	0.18	Non-Significant	0.22

**Table 14:** Confidence intervals of the *medians* of *Precision*, *Recall*, *F1-Score*, and *Accuracy* for 250, 500, 750, and 1000 iterations of bootstrap with 90%.

Metric	Iterations	Std. Dev. of Median Differences	Confidence Interval	Significance
**Precision**	250	0.10	[−0.1, 0.26]	Non-Significant
	500	0.09	[−0.06, 0.23]	Non-Significant
	750	0.09	[−0.06, 0.23]	Non-Significant
	1000	0.09	[−0.06, 0.23]	Non-Significant
**Recall**	250	0.16	[0.07, 0.57]	Significant
	500	0.16	[0.07, 0.57]	Significant
	750	0.16	[0.07, 0.57]	Significant
	1000	0.15	[0.07, 0.57]	Significant
**F1-Score**	250	0.11	[0.05, 0.39]	Significant
	500	0.11	[0.03, 0.39]	Significant
	750	0.11	[0.03, 0.38]	Significant
	1000	0.11	[0.03, 0.38]	Significant
**Accuracy**	250	0.08	[−0.05, 0.24]	Non-Significant
	500	0.08	[0, 0.25]	Non-Significant
	750	0.08	[0, 0.24]	Non-Significant
	1000	0.08	[0, 0.24]	Non-Significant

## Data Availability

The original data presented in the study are openly available in Zenodo at https://doi.org/10.5281/zenodo.13896006.
